# Genome-Wide Identification and Abiotic Stress Expression Analysis of *CKX* and *IPT* Family Genes in Cucumber (*Cucumis sativus* L.)

**DOI:** 10.3390/plants13030422

**Published:** 2024-01-31

**Authors:** Yang Xu, Shengxiang Ran, Shuhao Li, Junyang Lu, Weiqun Huang, Jingyuan Zheng, Maomao Hou, Fenglin Zhong

**Affiliations:** 1 College of Horticulture, Fujian Agriculture and Forestry University, Fuzhou 350002, China; xu10022020@163.com (Y.X.); sxran0601@163.com (S.R.); jylu@fafu.edu.cn (J.L.); 2 Fujian Seed Station, Fuzhou 350003, China; hwq36@126.com; 3 Vegetable Research Institute, Hunan Academy of Agricultural Sciences, Changsha 410128, China; zhengjingyuan2004@163.com

**Keywords:** cucumber, *CsCKXs*, *CsIPTs*, growth and development, abiotic stress response

## Abstract

Cytokinins (CKs) are among the hormones that regulate plants’ growth and development, and the *CKX* and *IPT* genes, which are CK degradation and biosynthesis genes, respectively, play important roles in fine-tuning plants’ cytokinin levels. However, the current research on the function of *IPT* and *CKX* in cucumber’s growth, development, and response to abiotic stress is not specific enough, and their regulatory mechanisms are still unclear. In this study, we focused on the *IPT* and *CKX* genes in cucumber, analyzed the physiological and biochemical properties of their encoded proteins, and explored their expression patterns in different tissue parts and under low light, salt stress, and drought stress. Eight *CsCKX* and eight *CsIPT* genes were identified from the cucumber genome. We constructed a phylogenetic tree from the amino acid sequences and performed prediction analyses of the cis-acting elements of the *CsCKX* and *CsIPT* promoters to determine whether *CsCKXs* and *CsIPTs* are responsive to light, abiotic stress, and different hormones. We also performed expression analysis of these genes in different tissues, and we found that *CsCKXs* and *CsIPTs* were highly expressed in roots and male flowers. Thus, they are involved in the whole growth and development process of the plant. This paper provides a reference for further research on the biological functions of *CsIPT* and *CsCKX* in regulating the growth and development of cucumber and its response to abiotic stress.

## 1. Introduction

Cytokinins (CKs) play a crucial role in controlling the growth and development of plants, with a wide range of biological effects. They can be obtained in synthetic form or isolated from plants. As phytohormones, they play important regulatory roles in many plant development and abiotic stress responses [1,2,3]. Many reports have suggested that cytokinins exert stimulatory or inhibitory functions in different developmental processes, such as root growth and branching, control of root tip dominance, and chloroplast development [4]. Since cytokinins are mostly synthesized in the roots and stems of plant bodies, they also promote cell proliferation in stems, which includes the activity of apical and axillary meristematic tissues [5]. At the same time, they are important in the regulation of leaf phloem [6], the regulation of cell division and differentiation during photomorphogenesis in unfolding leaves [7], pistil development, female gametophyte development [8], and the development of the vascular formation layer [9,10]. Furthermore, these hormones also function in the distribution of biomass [11] and the reaction to environmental stimuli [12,13,14].

Isopentenyl transferases (IPTs) play a key role in the production of cytokinins in plants, utilizing both the methylerythritol phosphate (MEP) and mevalonate (MVP) pathways [12,15,16]. Further studies have shown that catalysis occurs both de novo and via tRNA degradation. CKs’ biosynthesis and degradation are catalyzed by IPTs and cytokinin oxidases/dehydrogenases (CKXs), respectively, which are responsible for regulating endogenous CK levels [17]. IPT is the first rate-limiting enzyme for CK signaling, which is a key function in plant development and abiotic stresses; it was first identified in *Agrobacterium rhizogenes* [18,19], where it can be expressed throughout the plant and exists in the form of ATP/ADP and tRNA IPTs, and IPT enzymes are involved in the first and critical step of cytokinin synthesis. In plants, IPTs mediate two types of CK synthesis pathways: the ATP/ADP pathway and the tRNA pathway. Cytokinin oxidases (CKXs) are responsible for the irreversible breakdown of cytokinins in plants [20]. This is encoded by a small family of genes, and the substrate specificity of IPTs has been shown to vary depending on origin and species [12]. In the same manner, CKX enzymes exhibit their own unique substrate specificity, with distinct patterns of expression both spatially and temporally [21]. Cytokinins have a significant function in plants’ ability to adjust to intricate variations in their environment [22], and the *IPT* and *CKX* gene families have been recognized and replicated in various plant species. In *Arabidopsis thaliana*, the *atipt8* mutant and the *atipt1*, *3*, *5*, *7* quadruple mutant were found to enhance salt and drought tolerance in plants [23,24]. In tobacco, a stress-inducible promoter was found to drive the soil bacillus *IPT* gene, which was inserted into the tobacco genome, increasing the concentration of active cytokinins in the plant and enhancing drought tolerance [25]. Subsequently, similar phenomena were observed in transgenic rice and poplar, where stress-inducible promoters were found to drive the *IPT* gene [13,26]. Cold-inducible promoter-driven *IPT* gene expression was found to improve sugarcane’s tolerance to low temperatures [21]. Root-specific/constitutive promoters drive *CKX* gene expression, which reduces cytokinin contents and enhances drought tolerance in plants [27,28]. More importantly, stress can repress the activity of the root-specific promoter (WRKY6). Therefore, the root-specific promoter WRKY6 drives *CKX* gene expression, which reduces the cytokinin contents in roots, promotes root growth, and enhances drought tolerance. The *IPT* and *CKX* genes were also found to be involved in the resistance of maize and soybean to drought and salt stress. These findings indicate that the *IPT* and *CKX* genes have significant functions in controlling the ability of plants to adapt to challenging conditions. These investigations offer novel perspectives for the development of stress-tolerant plants through breeding.

Cucumber (*Cucumis sativus* L.) is a significant vegetable crop across the globe and is a vital part of meeting the global demand for vegetables. During their production process, cucumbers are often subjected to low temperatures, low light, salinity, drought, and other stresses, resulting in a decline in yield and quality. At present, there is insufficient research specifically examining how *IPT* and *CKX* contribute to the growth and development of cucumbers under stress conditions, and the underlying regulatory mechanisms remain unclear. To address this gap, our study focused on several key goals: identifying all *IPT* and *CKX* genes in cucumbers; analyzing the physiological and biochemical properties of proteins encoded by these genes; investigating the expression patterns of these genes in different cucumber tissues and their responses to low light, salt, and drought stresses; and examining how these mechanisms contribute to breeding efforts to overcome the effects of abiotic stress and exogenous phytohormones. Our aim is to provide a reference point for future research into the biological functions of *CsIPT*/*CsCKX* in regulating cucumber’s growth and development, and in responding to adverse conditions.

## 2. Experimental Materials and Methods

### 2.1. Plant Material and Growing Conditions

The experiment was conducted at Fujian University of Agriculture and Forestry, using Zhongnong 26 cucumber as the test material. Cucumber seeds were germinated at room temperature and sown in 32-hole trays with a substrate for seedling development. Upon reaching 2 leaves and 1 heart, uniformly grown cucumber seedlings were selected for hydroponic treatment by planting them in 3.5 L black plastic containers with Yamazaki’s nutrient solution (recorded as day 0). The greenhouse environment was maintained at a temperature of 26 ± 4 °C, with a 12 h/12 h photoperiod, approximately 55 ± 10% relative humidity, and light intensity of 200 µmol/m^−2^/s^−1^ PPFD with white LED lights (7:00–19:00). Treatments were carried out after 6 d of pre-cultivation, and 3 treatments were set up: T1: 65 µmol/m^−2^/s^−1^ PPFD far-red light (to simulate the effect of shade); T2: 20% PEG; T3: 200 mmol/L NaCl. Different tissue parts of the cucumbers were collected after 0, 1, 3, and 8 h of treatment, and the samples were placed in liquid nitrogen and immediately stored in a refrigerator at −80 °C. The experiment was replicated three times, with five plants from each treatment group.

### 2.2. Identification and Physicochemical Properties of CsCKX and CsIPT Genes in the Cucumber Genome

Based on *Arabidopsis IPT* gene sequences and *CKX* gene sequences (see Attachment 1 for details), Blast searches were performed in the Cucumber Genome Database (http//cucumber.genomics.org.cn/page/cucumber/index.jsp, accessed on 4 July 2023), and combined with the retrieved gene annotations, 8 *CsIPT* and 8 *CsCKX* candidate genes were identified, with a critical expectation of 100. Cucumber (*Cucumis sativus* L.) CsCKX and CsIPT candidate proteins were searched by Blast with TBtools. Arabidopsis protein data were obtained from TAIR (https://www.arabidopsis.org/, accessed on 5 July 2023), and cucumber protein data were obtained from the Cucurbit database (http://cucurbitgenomics.org/, accessed on 5 July 2023). Hidden Markov model (HMM) files for *CsIPT* (PF01715)/*CsCKX* conserved structural domains (PF15628) were downloaded from the Pfam database (http://pfam.xfam.org/search, accessed on 5 July 2023), using PF01715/PF15628 as templates. The hmmsearch program of TBtools was used to search for proteins containing this conserved structural domain in the cucumber proteome. The search results of the above methods were de-duplicated and analyzed using the NCBI Conserved Domains Database (CDD; http://www.ncbi.nlm.nih.gov/cdd, accessed on 5 July 2023), and the cucumber protein data were obtained from the Cucurbit database (http://cucurbitgenomics.org/, accessed on 5 July 2023). These data were validated against the screened sequences, where the E-value was taken as 10^−4^. Protein identification and analysis tools on the ExPASy program (https://web.expasy.org/protparam/, accessed on 12 July 2022) were used to identify CsCKX and CsIPT proteins for physicochemical properties, including chemical properties, amino acid number (aa), protein size, isoelectric point (pI), molecular weight (MW), instability index, aliphaticity, and hydrophilicity prediction. The subcellular localization of CsCKX and CsIPT proteins was predicted using Plant-mPLoc (http://www.csbio.sjtu.edu.cn/bioinf/plant-multi/, accessed on 22 July 2022) [29].

### 2.3. Protein Structure Analysis of CsCKX and CsIPT

The ExPASy website (https://swissmodel.expasy.org/interactive, accessed on 11 July 2023) facilitated the analysis of the proteins’ secondary structure and the prediction of their tertiary structure. The necessary parameters were used to construct protein tertiary structure models.

### 2.4. Chromosome Localization and Collinearity Analysis

The Chinese long v3 gff3 file was downloaded from the Cucumber Genome Database to obtain the chromosome number, length, and the start and end positions of *CsCKX* and *CsIPT* gene family members on the chromosomes, and the chromosome distribution of the *CsCKX* and *CsIPT* genes was mapped by using TBtools (accessed on 3 June 2022). Homology searches were performed using the multicollinear scanning toolkit MCScanX to analyze gene duplication events using the default values. Using BLASTp, the cucumber genome’s protein-coding genes were compared to both its own genome and the Arabidopsis genome. The search threshold was set to an E-value less than 10^−5^, and no modifications were made to the default parameters for the other variables. Subsequently, TBtools (accessed on 29 July 2022) was used to highlight the collinear relationships within the identified *CsIPT*/*CsCKX* gene families and with genes homologous to these gene families in other species.

### 2.5. Phylogenetic Relationship Analysis

Phylogenetic trees were estimated using the MEGA 7.0 program, using the neighbor-joining (NJ) method with 1000 replicates for bootstrap analysis. PFAM structural domains and signal peptides of the *IPT* and *CKX* genes were obtained from the PFAM database. The exon/intron organization of the *CsIPT* and *CsCKX* genes was determined by comparing exon positions and gene lengths using the GSDS gene structure website (http://gsds.cbi.pku.edu.cn, accessed on 3 June 2022).

### 2.6. Analysis of Conserved Motifs and Gene Structure of Members of the CsCKX and CsIPT Families

We used the MEGA 7.0 method to construct phylogenetic trees for the *IPT*/*CKX* gene families of cucumber, *Arabidopsis thaliana*, melon, and non-bearing cabbage. The trees were built with 1000 bootstrap values and multiple sequence alignment. From these trees, we selected a stable minimum neighborhood tree to represent their evolutionary relationships. To enhance the visual presentation of the tree, we utilized iTOL (https://itol.embl.de/, accessed on 23 July 2022).

### 2.7. Analysis of Cis-Acting Elements

To analyze the cis-acting elements contained in the promoter regions of *CsCKX* and *CsIPT* family members, we obtained 2000 bp DNA sequences upstream of their start codons using TBtools software. The PlantCARE website (http://bioinformatics.psb.ugent.be/webtools/plantcare/html/, accessed on 5 June 2022) was used to analyze the cis elements.

### 2.8. Expression Analysis of Gene Expression Levels under Multiple Stresses in Different Tissues and Organs

To investigate the gene-specific expression of *CsCKX* and *CsIPT* genes in different tissues of cucumber, firstly, we obtained their gene sequences from the Cucumber Genome Database (http://cucurbitgenomics.org/, accessed on 5 June 2022) and performed RNA extraction on different tissues (e.g., tendril, root, leaf, stem, flower, fruit) for quantitative analysis. The log_2_ function was employed to convert the data, and TBtools was utilized to create an expression heatmap for the *CsCKX* and *CsIPT* genes to further investigate the changes in gene expression levels under multiple stresses in different organs of cucumber.

### 2.9. RNA Extraction and Real-Time Fluorescence Quantitative qPCR Analysis

Quantitative analysis was performed using a kit (see Attachment 2 for details). The primers and internal reference genes used for qPCR amplification are listed in Table 1. Relative gene expression was calculated using the 2^−ΔΔCT^ method. Sample analysis was carried out using three biological replicates.

### 2.10. Data Analysis

Data were processed and plotted using SPSS (IBM^®^SPSS^®^ Statistical Version 24) and GraphPadPrism8 (San Diego, CA, USA).

## 3. Results

### 3.1. Identification of CsCKX and CsIPT Genes in the Cucumber Genome and Characterization of Their Physicochemical Properties

Arabidopsis AtIPT and AtCKX proteins were used as query sequences [30,31] to identify *CsIPT*/*CsCKX* genes in the cucumber genome. After screening, eight *CsIPT* genes and eight *CsCKX* genes with confirmed conserved structural domains were identified and annotated (Table 2), and the genes were named according to their homologues in Arabidopsis. As shown in Table 2, the eight *CsCKX* genes have amino acids ranging from 516 to 699 aa, relative molecular weights ranging from 57.37 to 79.32 kD, and isoelectric points between 5.27 and 9.1, with CsCKX2, -5, -7, and -8 being acidic proteins. The stability coefficients were less than 40, except for CsCKX6, which was found to be a stable protein. In addition, all members are hydrophilic proteins except for CsCKX3, which is hydrophobic. Subcellular localization prediction of the proteins of CsCKXs revealed that, except for the proteins of CsCKX1, -3, and -4, which are located in vesicles, the rest are located in the cytoplasmic matrix. Subsequently, the physicochemical properties of the eight CsIPTs were analyzed, and it was found that they had 320 to 474 aa, relative molecular weights from 35.8 to 53.14 kD, and isoelectric points ranging from 5.71 to 9.0, with CsIPT4, -6, -7, and -8 being acidic proteins. Except for CsIPT1, -2, and -8, which were found to be unstable proteins, the rest have a stability factor less than 40, indicating that they are stabilizing proteins. In addition, all of these protein members are hydrophilic proteins. Prediction of subcellular localization of the CsIPT proteins revealed that the CsIPT1, -3, and -6 proteins are located in chloroplasts, the CsIPT2 and -5 proteins are located in chloroplasts and mitochondria, CsIPT4 is located in mitochondria, CsIPT7 is located in chloroplasts, mitochondria, and peroxisomes, and CsIPT8 is located in chloroplasts, cytoplasm, mitochondria, and peroxisomes.

### 3.2. Protein Structure Analysis of CsCKX and CsIPT

The sequence and structure of a protein can reflect its function and provide the basis for its tertiary structure model. We utilized SOPMA online (https://npsa-prabi.ibcp.fr/cgi-bin/npsa_automat.pl?page=npsa%20_sopma.html, accessed on 1 June 2022)to predict the secondary structure of CsCKX and CsIPT proteins, aiming to gain a better understanding of their structural characteristics. As shown in Table 3, all CsCKX and CsIPT protein structures consisted of *α*-helices, *β*-folds, extended strands, and irregular coils, with the proportion of *α*-helices in CsCKXs ranging between 31.44% and 35.85%, the proportion of *β*-folds ranging between 4.94% and 7.13%, the proportion of extended strands ranging between 17.05% and 20.66%, and the proportion of irregular coils ranging between 40.77% and 45.52%. The proportion of *α*-helices in CsIPTs ranged between 43.33% and 56.35%, the proportion of *β*-folds ranged between 4.43% and 8.59%, the proportion of extended strands ranged between 10.34% and 13.33%, and the proportion of irregular coils ranged between 25.39% and 36.97%. An analysis of the secondary structures showed that there were differences between CsCKX and CsIPT proteins, but the differences were smaller for CsIPTs than for CsCKXs.

**Figure 1:**
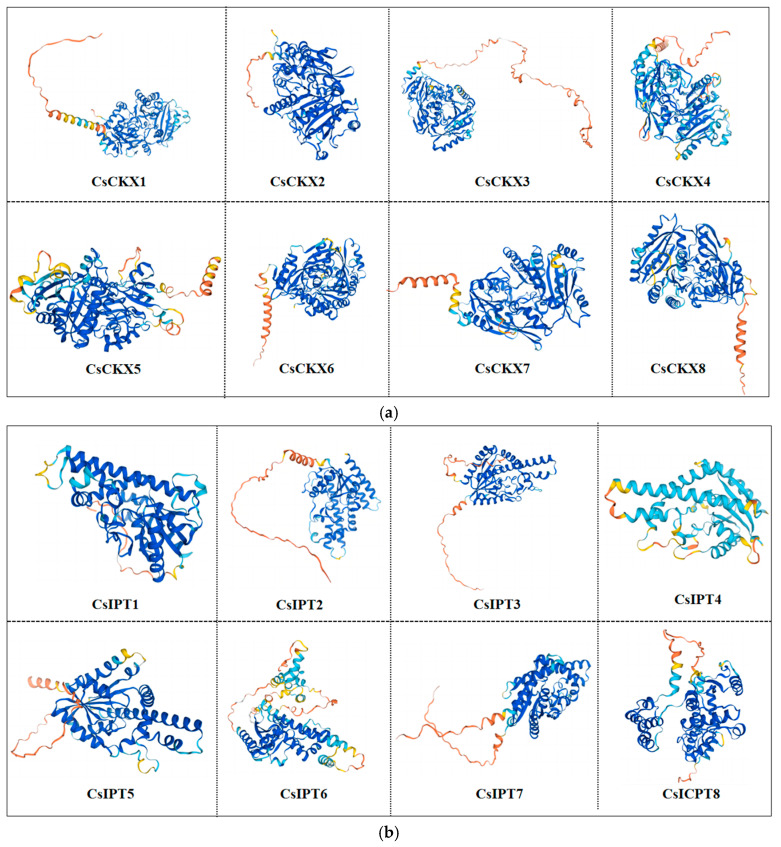
Tertiary structure of CsCKX (**a**) and CsIPT (**b**) proteins.

The function of a gene is linked to the structure of the protein that it encodes. Homology modeling is a technique that relies on the amino acid sequence of a protein to create a 3D structure for a specific protein of interest by utilizing the 3D structure of a closely related protein. Using the homology modeling method, we successfully predicted the 3D structures of the eight proteins of CsIPT/CsCKX (Figure 1). The modeling results reveal that the GMQE values of the CsIPT/CsCKX protein sequences were all in close proximity to 1, demonstrating a strong uniformity among the proteins and a significant level of certainty in the predictions.

### 3.3. Chromosomal Placement and Collinearity Analysis of CsCKXs and CsIPTs

**Figure 2:**
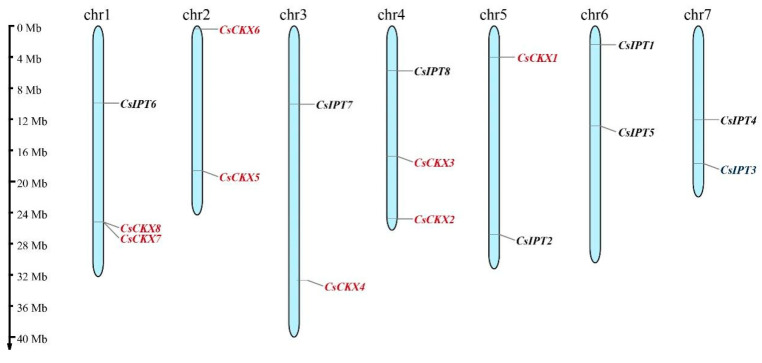
Location of *CsCKX* and *CsIPT* gene family members on chromosomes. The scale bar on the left was used to estimate the length of the chromosomes. *CsCKX* and *CsIPT* gene family members are numbered in order from 1 to 8, with *CsCKXs* marked in red and *CsIPTs* in black.

The locations of the *CsCKX* and *CsIPT* gene families on chromosomes in relation to covariance within species are shown in Figure 2. According to the cucumber genome annotation file, we identified eight members of the *CsCKX* gene family and eight members of the *CsIPT* gene family. These genes are located on different chromosomes: the *CsCKX* genes are found on chromosomes 1–5, and the *CsIPT* genes are found on chromosomes 1, 3, 4, 5, 6, and 7 (Figure 2). Among the eight *CsIPT* family members, only *CsIPT3* and -4 are located in regions far from the telomeric region.

**Figure 3:**
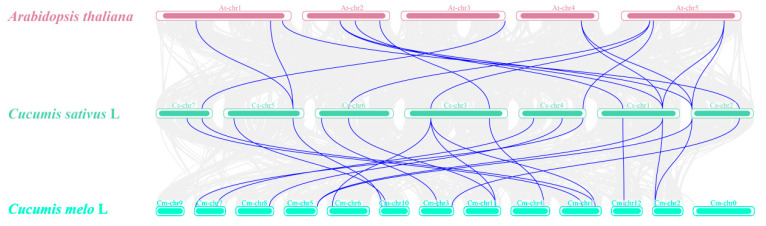
Collinearity between *CsCKX* and *CsIPT* gene family members was analyzed in cucumber, Arabidopsis thaliana, melon, and other species. The chromosomes of these plants are marked with different colors, and the chromosome is indicated above each chromosome. Collinear relationships between *CsCKX* and *CsIPT* gene family members in different species are connected by lines. Homologous *CsCKX* and *CsIPT* gene pairs are represented by blue lines.

To further infer the phylogenetic mechanisms of the *CsCKX* and *CsIPT* families and identify the collinear relationships between the eight genes of these families and those of other species, we performed collinearity analysis of the *CsCKX* and *CsIPT* family members of *Arabidopsis thaliana*, melon, and cucumber. As shown in Figure 3, a strong collinear region was observed between the dicotyledonous and Cucurbitaceae plants. Among these three plants, cucumbers and melons are the most closely related due to their genetic background as members of the Cucurbitaceae family. The analysis of genetic relationships and gene functions of various species can be facilitated by comparing the families of *CsCKX* and *CsIPT* with those of other species, thus offering a valuable research reference.

### 3.4. Analysis of Phylogenetic Relationships

**Figure 4:**
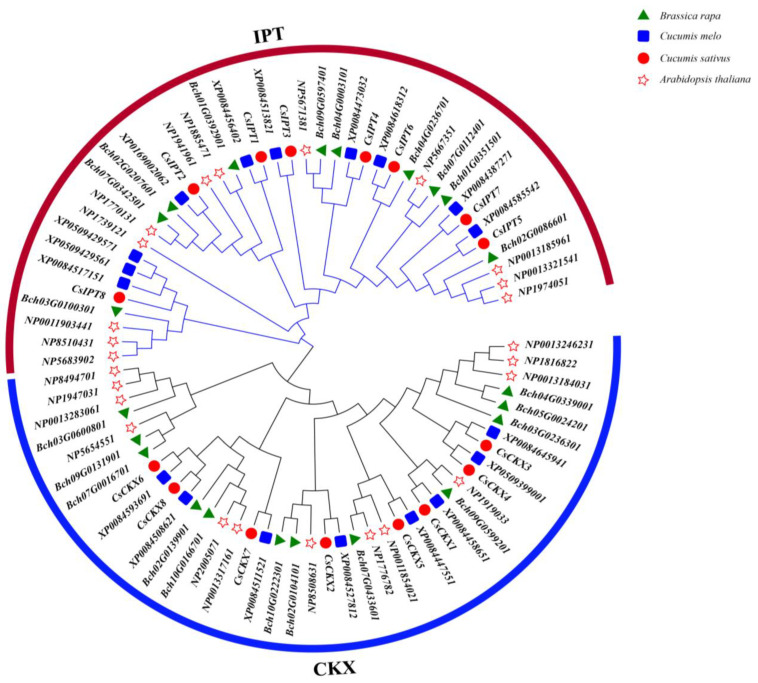
Phylogenetic tree: different species are labeled with different shapes. Circles represent cucumber, pentagrams represent Arabidopsis thaliana, squares represent melon, triangles represent Brassica rapa, blue lines represent *CKX*, and red lines represent *IPT*.

In order to examine how *CsCKX* and *CsIPT* genes are related to other similar genes, we built a phylogenetic tree using the amino acid sequences of several identified genes. This included 13 *AtCKX* genes and 12 *AtIPT* genes, 10 *CmCKX* genes and 8 *CmIPT* genes, 10 *BrCKX* genes and 12 *BrIPT* genes, and 8 *CsCKX* genes and 8 *CsIPT* genes (Figure 4). By analyzing the phylogeny of homologous genes in each species, it was found that cucumber and melon showed a close evolutionary relationship for both *CKX* and *IPT* genes, which might be related to their belonging to the cucurbit family. Considering the frequency of members of the same subfamily performing similar tasks enhances our understanding of the potential biological functions of the *CsCKX* and *CsIPT* families.

### 3.5. Conserved Motif and Gene Structure Analyses of Cucumber CsCKX and CsIPT Family Members

**Figure 5:**
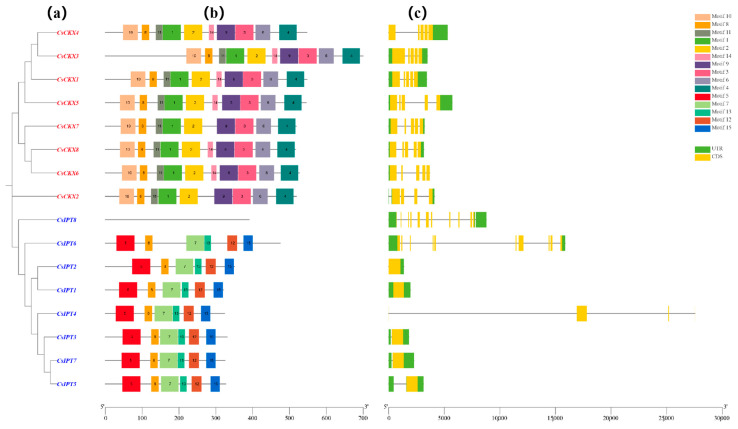
The members of the *CsCKX* and *CsIPT* gene families exhibit a conserved gene structure and motifs. The gene family members are presented in a phylogenetic tree (**a**) showing the relationships between *CsCKXs* and *CsIPTs*. The distribution order of the conserved motifs in *CsCKXs* and *CsIPTs* is shown in (**b**), with different motifs indicated by different colored boxes (**c**). Additionally, the distribution of UTR and CDS in *CsCKXs* and *CsIPTs* is represented by green and yellow colors, respectively. The scale bar at the bottom allows for the comparison of gene and protein lengths.

We used MEGA-7 to construct a phylogenetic tree consisting exclusively of genes belonging to the *CsCKX* and *CsIPT* families, and the results were consistent with the previous evolutionary tree. The gene members of the *CsCKX* and *CsIPT* families were then analyzed for conserved motifs and gene structures based on this tree (Figure 5).

In the gene structure analysis, the eight *CsCKX* genes contained five exons each, and all of them contained two introns except for *CsCKX1* and -*2*, which contained one intron. Among the *CsIPT* gene family members, *CsIPT1*, -*3*, and -*7* contained one exon and two introns, *CsIPT2* contained one exon and one intron, *CsIPT4* contained only two introns, *CsIPT*5 contained one exon and three introns, *CsIPT6* contained ten exons and two introns, and *CsIPT8* contained eleven exons and three introns. During the investigation of *CsCKX* conserved motifs, we discovered and assigned names to 10 conserved motifs: motifs 1, 2, 3, 4, 6, 8, 9, 10, 11, and 14. The analysis revealed that all genes in the group contained these 10 conserved motifs arranged in an identical sequence, except for *CsCKX2* and -*7*, where motif 14 was absent. Examining *CsIPT*, six conserved motifs were identified, except for *CsIPT*8, which did not have any motifs; they were named motifs 5, 7, 8, 12, 13, and 15.

### 3.6. Analysis of Cucumber CsCKX and CsIPT Family Members to Predict Their Cis-Acting Elements

**Figure 6:**
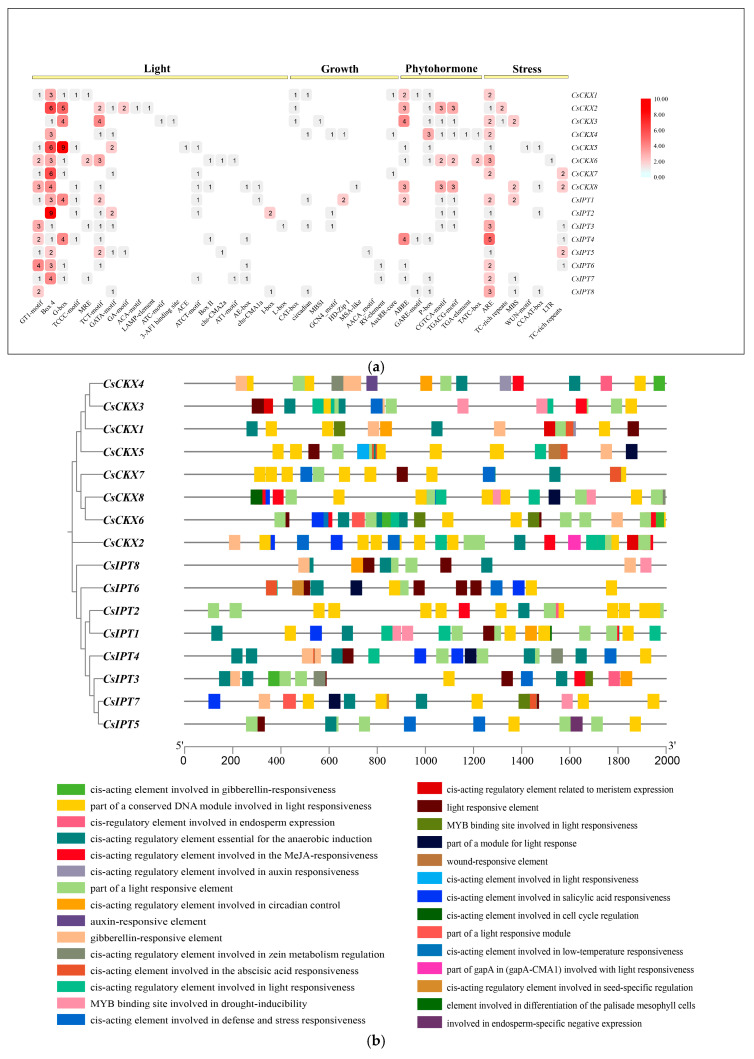
Analysis of promoters of *CsCKXs* and *CsIPTs* to predict the presence of cis-acting elements: (**a**) For each promoter, the number of detected cis-acting elements was counted, and they were classified into four types. (**b**) TBtools was used to visualize response elements in promoters of *CsCKXs* and *CsIPTs*, showing their location and type.

In this study, we utilized the sequences located 2000 bp before each gene to forecast cis-acting elements within the eight genes from the *CsCKX* and *CsIPT* families, emphasizing their significance in gene transcription (Figure 6). Figure 6a,b show the enrichment and location of cis-acting elements in the promoters of the *CsCKX* and *CsIPT* gene families. The findings indicate that both gene families contain significant numbers of cis-acting elements, primarily related to the responses to abiotic stress (58), light (178), growth and development (23), and phytohormones (66). Notably, numerous elements associated with light response, such as G-box, Box-4, and GT1-motif, are prevalent in most *CsCKX* and *CsIPT* genes, suggesting that light may play a role in inducing or repressing the expression of these genes.

All *CsCKX* family members contain Box-4, and all family members except for *CsCKX8* contain ARE. Similarly, all *CsIPT* family members contain ARE, and all members except for *CsIPT8* contain Box-4. The largest proportion of cis-acting elements in the promoter regions of *CsCKX* and *CsIPT* family members consists of light-responsive elements, totaling 178. The smallest proportion of elements consists of those associated with growth and development, with 23. *CsCKX6* and -*7*, along with *CsIPT4*, lack any elements related to growth and development.

### 3.7. Analysis of CsCKX and CsIPT Gene Expression in Different Tissues and Organs of Cucumber

**Figure 7:**
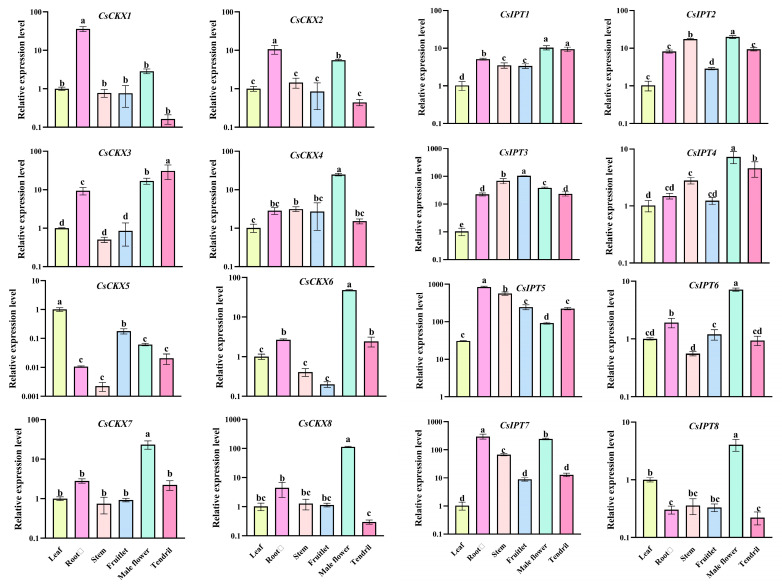
Analysis of *CsCKX* and *CsIPT* gene expression in different tissues and organs of cucumber. The data are expressed as the mean ± SD (*n* = 3 biological replicates) and are referenced to the mean value of actin gene expression, which is the average of 2^−ΔΔCT^_,_ The letters stand for significance, *p*-value < 0.05.

We determined the expression of each of the eight genes of *CsCKX* and *CsIPT*—i.e., *CsCKX1*–*CsCKX8* and *CsIPT1-CsIPT8*—in different tissues, and the results are shown in Figure 7. Among the eight *CsCKX* genes, they were expressed at high levels in the root system and male flowers, and *CsCKX8* was expressed at the highest level, which may be related to the expression of cytokinins in the root system and may also be involved in the opening of the flowers. Among the eight *CsIPT* genes, the trend of expression was similar, except for *CsIPT8*. It was found through the data that, except for the high level of the expression of the higher expression of the *CsIPT 1*, *2*, *3*, *5*, and -*7* genes may be related to the expression of cytokinin in the root system, indicating that these genes are involved in the whole growth and development process of the plant.

### 3.8. Expression Pattern of CKX/IPT Genes in Cucumber under Abiotic Stresses

**Figure 8:**
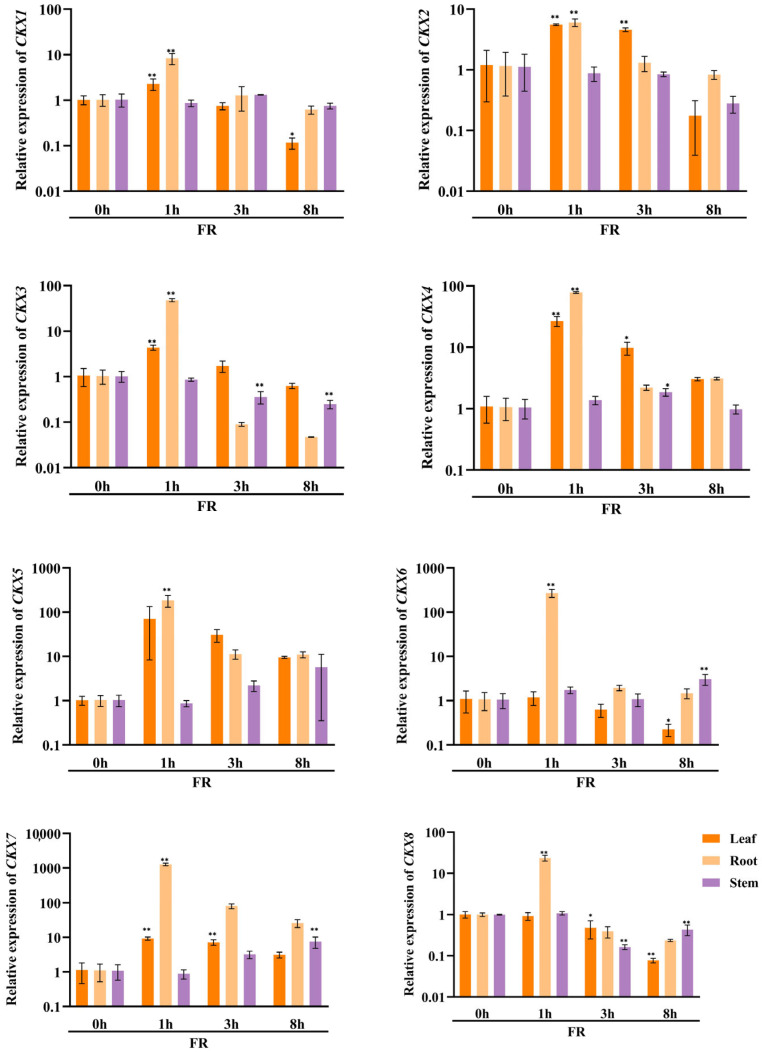

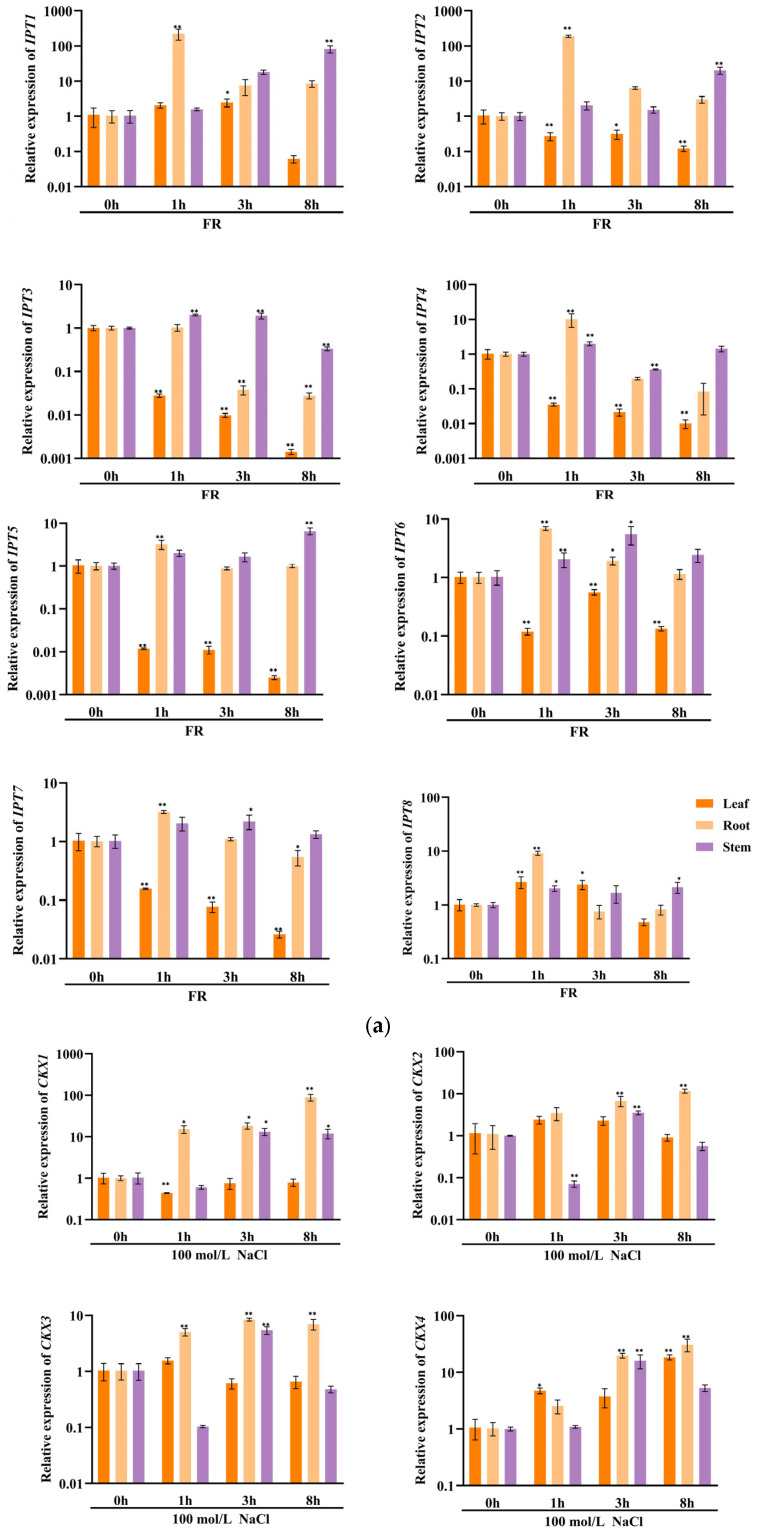

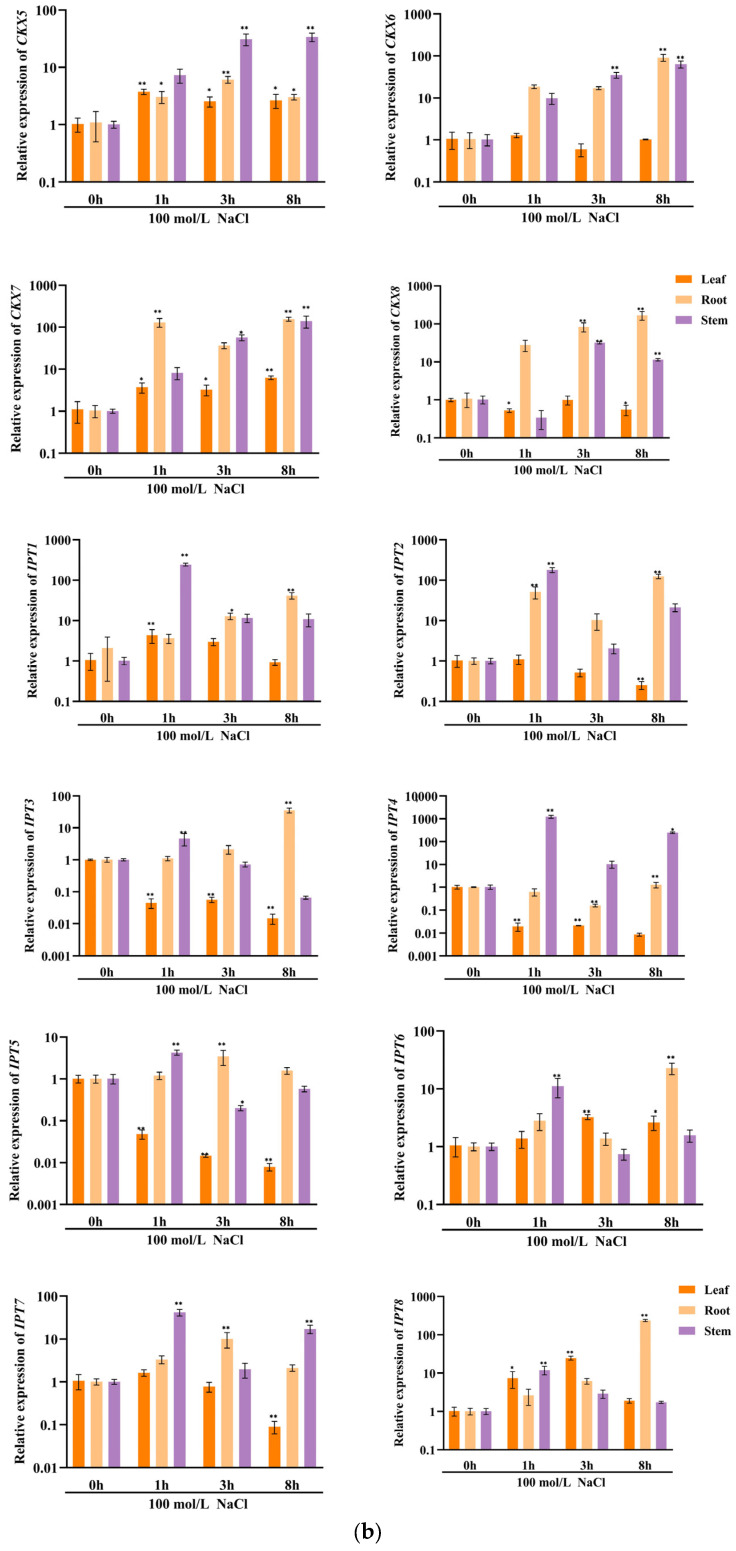

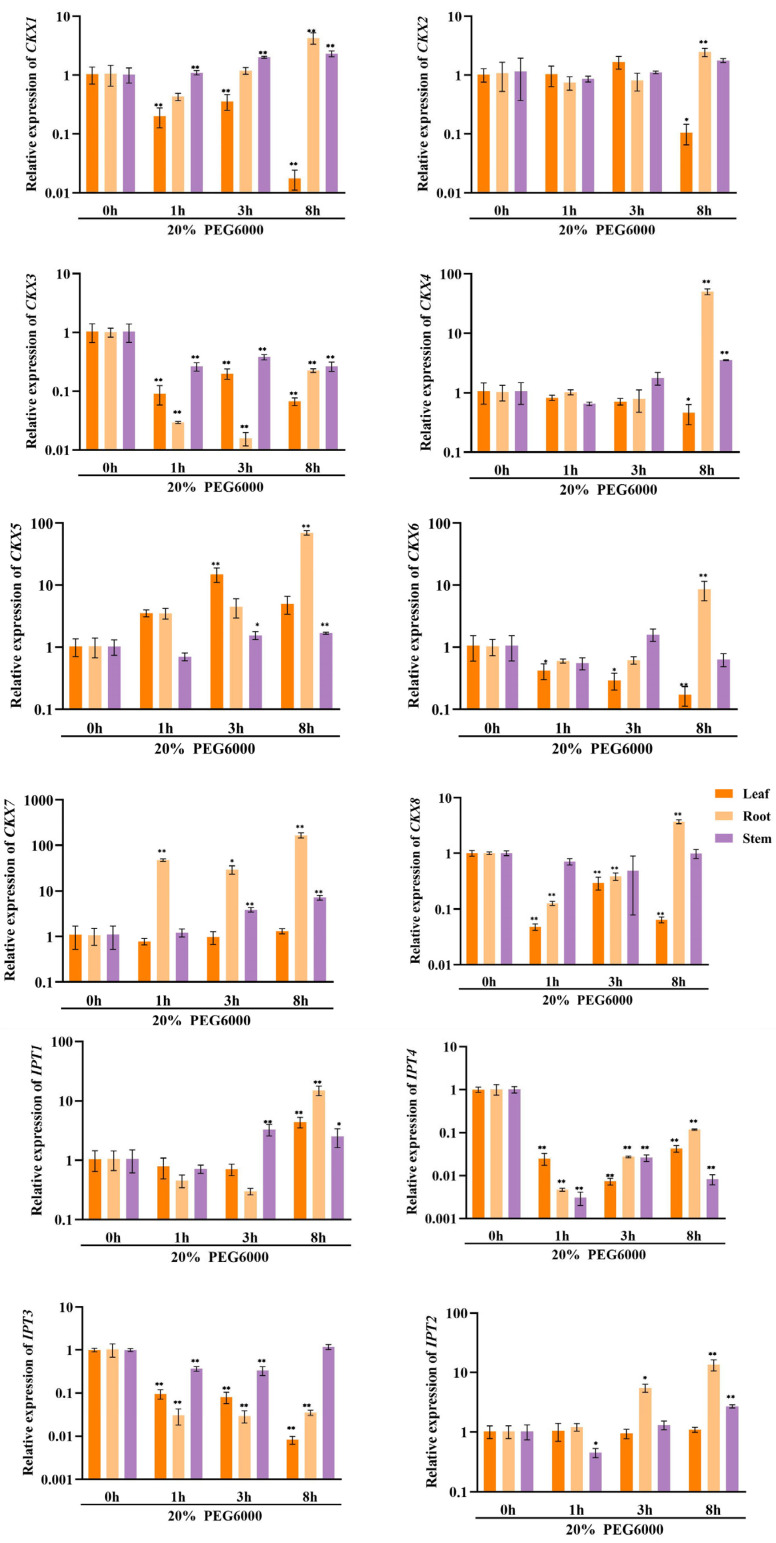

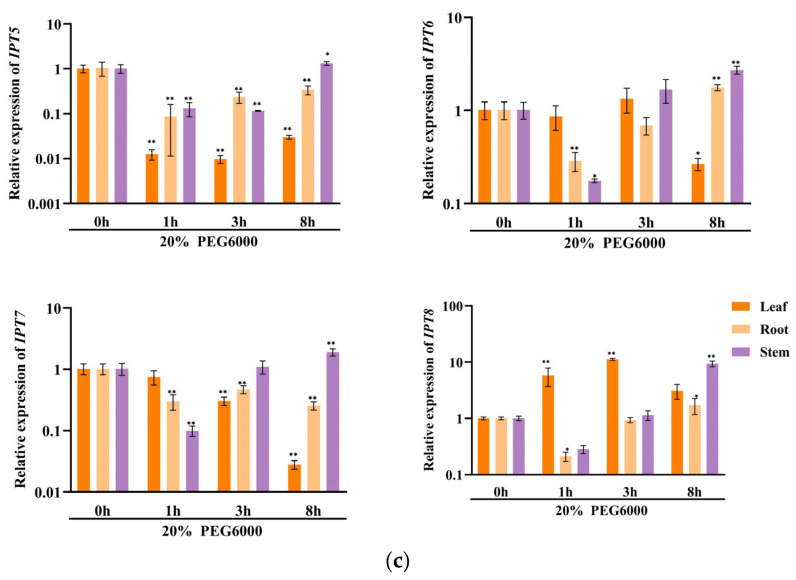
The relative expression levels of *CsCKX* and *CsIPT* genes at different timepoints (0, 1, 3, and 8 h) under abiotic stress. The bar graphs represent the relative expression of *CKX*/*IPT* genes in cucumber subjected to far-red light (**a**), salt stress (**b**), and drought (**c**). The data are presented as the mean and standard deviation (*n* = 3 biological replicates), and the mean expression of the actin gene was used as a reference. The value was calculated as the mean of 2^−ΔΔCT^; * indicates a significant difference (*p*-value < 0.05), and ** indicates a highly significant difference (*p*-value < 0.01).

In order to investigate the potential involvement of *CKX/IPT* genes from cucumber in various stress responses, we employed qRT-PCR to examine their expression levels. As shown in Figure 8a, the expression of all *CsCKX* members reached the highest levels after 1 h of FR treatment, with the root system showing the highest levels. However, as time progressed, expression among the members started to decline. The trend of *CsIPT* members under FR treatment was broadly similar to that of *CsCKX* members, except for *CsIPT6*, whose expression in leaves reached a peak at 3 h. The expression of the remaining members in leaves showed a decreasing trend. As shown in Figure 8b, following the application of NaCl, there was a gradual decrease in the expression of *CsCKX* members in leaves over time, except for *CsCKX4* and -*8*, which decreased in leaves and were elevated in the root system. Conversely, the expression in stems increased after NaCl treatment, with *CsIPT4* displaying the highest expression level, reaching a peak at the 1 h mark. Surprisingly, following PEG treatment (Figure 8c), the expression levels of *CsCKX* members in the root system peaked after 8 h. In the leaves, the expression of *CsCKX1*, -*2*, -*3*, -*5*, and -*8* initially increased and then decreased over time, reaching a peak at 3 h. Additionally, the expression of *CsIPT* members in stems reached a maximum after 8 h, with a similar pattern seen among the members.

## 4. Discussion

*IPT* family genes have been shown to induce unisexual fruiting in tomatoes [32], and only the *CsIPT1* gene has been isolated from cucumber, but its specific gene function has not been analyzed and identified [33]. The ability to sequence the cucumber genome and create a database [34] has created an opportunity for comprehensive investigation into the molecular mechanisms behind unisexual nodulation in cucumbers. Gene family analysis has become an effective method for better understanding genes’ structure, function, and evolution.

Cytokinin dehydrogenases have the ability to control cytokinin levels and are crucial for maintaining a balance between the synthesis and breakdown of cytokinins in plants [35,36]. Researchers have extensively studied the *IPT* and *CKX* gene families to understand their role and evolutionary history in various plant species. For instance, *Arabidopsis thaliana* has 7 *AtCKX* genes and 9 *AtIPT* genes; rice has 11 *OsCKX* and 10 *OsIPT* genes; soybean has 17 *GmCKX* and 14 *GmIPT* genes; cabbage has 12 *BrCKX* and 13 *BrIPT* genes; and maize has 13 *ZmCKX* and 8 *ZmIPT* genes [37,38,39,40,41,42,43]. Currently, the studies on *IPT*/*CKX* in cucumber’s growth, development, and responses to stress are not specific enough and have not clarified their regulatory mechanism(s). The primary goals of this research were to identify all *IPT* and *CK*X genes in cucumber, examine the physiochemical characteristics of the associated proteins, investigate the gene expression patterns in various cucumber tissues, and determine their responses to low light, salt, and drought stresses. Additionally, we aimed to uncover the mechanisms by which these genes cope with abiotic stresses and external phytohormones in order to facilitate breeding efforts. Ultimately, our findings will help in establishing a foundation for future investigations into the biological functions of *CsIPT*/*CsCKX* in cucumber’s growth, development, and responses to stressful conditions.

A phylogenetic tree was created in this research by using the amino acid sequences of 8 *CsCKX* and 8 *CsIPT* genes, 13 *AtCKX* and 12 *AtIPT* genes, 10 *CmCKX* and 8 *CmIPT* genes, and 10 *BrCKX* and 12 *BrIPT* genes that were detected in the cucumber genome (Figure 4). Phylogenetic analysis using protein sequences from multiple species revealed that cucumber and melon exhibited a significant evolutionary connection in terms of both *CKX* and *IPT* genes, suggesting a potential relationship within the cucurbit plant family. By considering the frequency of members of the same subfamily performing similar tasks, the *CsCKX* and *CsIPT* families can provide us with a deeper understanding of their potential biological activities.

The examination of the gene structure and conserved structural domains of *CsCKX* and *CsIPT genes* revealed that the eight members of the *CsCKX* gene family included two introns and five exons, with the exception of *CsCKX1* and -*2*, which had a single intron. The gene structure of the *CsIPT* gene family members was more complex and variable, and it was unstable compared with *CsCK*X. From the results, it can be seen that except for *CsCKX2* and -*7*, which lacked motif 14, all of the remaining member genes possessed all 10 conserved motifs, and the motifs were organized in an identical sequence. During the examination of the conserved motifs in *CsIPT*, with the exception of *CsIPT8*, which lacked any motifs, six conserved motifs were discovered and designated as motifs 5, 7, 8, 12, 13, and 15, respectively.

The growth and expression of *CsCKX* and *CsIPT* genes are also regulated by gene promoter activity [30]. The results of cis-acting element prediction analysis of *CsCKX* and *CsIPT* promoters showed that both gene families may be responsive to light, abiotic stresses, and different hormones. The analysis focused on their expression in various tissues and organs of cucumber (Figure 7). Upon analysis of the data, it was found that the expression of *CsCKX* and *CsIPT* genes was significantly higher in the root system and male flowers. It was concluded that the roots are the main site of cytokinin biosynthesis, and cytokinins synthesized by the roots not only regulate their own differentiation but are also transported to other parts of the plant to affect growth and development [44,45]. Therefore, their involvement extends throughout the entire growth and development of the plant. When the *CKX1* gene is overexpressed in regular maize plants, it can result in male sterility [46]. It is hypothesized that the high expression of *CsCKX4*, *CsCKX6*, *CsCKX7*, *CsCKX8*, *CsIPT1*, *CsIPT2*, *CsIPT4*, *CsIPT6*, *CsIPT7*, and *CsIPT8* genes in male flowers of cucumber ensures the normal growth and development of male flowers. The role of cytokinins in regulating leaf morphogenesis has also been demonstrated. In cucumber, *CsCKX5*, *CsIPT2*, *CsIPT3*, *CsIPT4*, and *CsIPT5* had the highest expression levels in both leaves and stems, and these two components are potentially significant in the local synthesis of cytokinins within the leaves and stems.

The results of qRT-PCR expression analysis of *CKX* and *IPT* family genes under FR, drought, and salt stress treatments (Figure 8) show that cucumber seedlings exhibited a similar expression pattern for most members under light quality treatment. All members of the *CsCKX* group reached their highest expression levels after 1 h of FR treatment, with the root system showing the highest levels. As time passed, the expression levels started to decline. The trend of all *CsIPT* members under FR treatment was broadly similar to that of *CsCKX* members, except for *CsIPT6*, for which the peak expression in leaves occurred at 3 h, after which the expression of all members gradually decreased. As shown in Figure 8b, after NaCl treatment, the expression of *CsCKX* members in leaves showed a decreasing trend over time, except for *CsCKX4* and *CsCKX8*, for which there was a decline in the trend and a significant increase in expression in the root system. The expression in stems was also upregulated following treatment with NaCl, and *CsIPT4* had the highest expression, which peaked at 1 h. Interestingly, after PEG treatment (Figure 8c), at 8 h, the members of *CsCKX* had their highest expression levels in the root system. The expression of *CsCKX1*, -*2*, -*3*, -*5*, and -*8* in leaves showed an initial increase followed by a decrease over time, with the peak occurring at 3 h. Interestingly, the expression of *CsIPT* members in the stems reached its highest values at 8 h, with a similar trend among the members.

In order to gain a deeper understanding of how the *CsCKX* and *CsIPT* families are phylogenetically related, and to determine the collinear relationships between the eight genes in each family and genes from other species, we conducted further analyses. We performed collinearity analysis of *CsCKX* and *CsIPT* family members in Arabidopsis, melon, and cucumber. As shown in Figure 3, a strong collinear relationship exists between dicots and melons. Of the three species, cucumber and melon are members of Cucurbitaceae and, thus, are the most closely related. A comparison of the *CsCKX* and *CsIPT* families with other species is useful in order to analyze genetic relationships and gene functions in multiple species.

## 5. Conclusions

During the course of this research, we successfully identified eight members from the *CsCKX* gene family, found on five different chromosomes, and eight members from the *CsIPT* gene family, distributed across six chromosomes, in cucumber. These genes play key roles in cytokinin biosynthesis and catabolic metabolic pathways. We performed a thorough and organized investigation of the *CsCKX* and *CsIPT* families, encompassing various aspects, including phylogenetic analysis, conserved structural domains, chromosomal localization, gene structure, and gene expression. In order to gain a deeper understanding of the underlying phylogenetic mechanisms, we performed a collinearity analysis of *CsCKX* and *CsIPT* family members from Arabidopsis, melon, and cucumber, the most closely related members of the Cucurbitaceae family. The results show that there was a highly significant correlation between the expression of *CsCKX* and *CsIPT* genes and promoter cis-elements under conditions of light, stress, and hormone treatments. In conclusion, this study’s findings offer new insights for further examination of the function of *CKXs* and *IPTs*, and for the development of the regulatory network in cucumbers. This provides a reference for related studies in other crops.

## Figures and Tables

**Table 1:** The gene-specific primers used.

Gene	Primer	
*CsTua*	F-CTCTCAACCCATTCTCTCTTGG	R-CGGTTGAGGTTCGAGTAGTTAG
*CsIPT1*	F-ATGCCTCCGCCGTCATCTCC	R-ACACCCGATTCCTAGACCCACAG
*CsIPT2*	F-TGAAACTGAAAGAGGCGGGTTGG	R-TTTCCCAAATCTCCTTCCTGTTCCG
*CsIPT3*	F-CCACCAGGAAGACTAACCACCAAC	R-GGACCGATCACGATCACAACCTTC
*CsIPT4*	F-AATCGTCGTCGGAGGATCAAACAAC	R-GCAGAGCAACATCGGTCCACAG
*CsIPT5*	F-TGTAGAGGAGGAGCCTGAGTTTCG	R-CCGACACGAACGAGTTGAGGATG
*CsIPT6*	F-TGGCTGCTGGATTGCTTGATGAG	R-AACTCCCGAACACCAATAGCTTGAC
*CsIPT7*	F-TCGATGCCACCGGAGTCATTTTG	R-GGCTACCGTCGCAGAGGAATTG
*CsIPT8*	F-TGGCCGTGTTCCGATAGTTTGTG	R-CTTCAGCAGCAATGTCTGGAGAGG
*CsCKX1*	F-TTCCTGCGGCAATACTACATCCATC	R-GTGAGTTGTGAACGAGGTCCCATC
*CsCKX2*	F-GATTGCTGAGTGGGCTTGGGTATG	R-CTTCCTCTTCCGCACGCTTCAC
*CsCKX3*	F-TGGCATTAGTGGGCAGGCTTTC	R-TCTCTGAACAGACAACCACCTCTCC
*CsCKX4*	F-TTCTCTTCAAGGTCAGGCACAAGC	R-ATCCACCCATGAAAGATTCCCACTG
*CsCKX5*	F-ACCCTGCCTCTGCCGATGAC	R-CCGACACCGTAAACCCACCATTC
*CsCKX6*	F-CATATAGCCGCAAGAGGACAAGGAC	R-CCACCGCCTACACGACACAAC
*CsCKX7*	F-CGTAATCTCACTTCTGGCGTCTTCC	R-AACACCTCCTCGTCGGGTATCAC
*CsCKX8*	F-TCTCCTCTGCTGCTACCGACTTC	R-TTGAAAGGAACAGAACGGGAGTTGG

**Table 2:** The general information of *CsCKX* and *CsIPT* family members.

Locus Name	Gene Name	Protein Name	Number of Amino Acids	Molecular Weight/kD	Isoelectric Point/pI	Instability Index	Aliphatic Index	Grand Average of Hydropathicity	Subcellular Localization
CsaV3_5G006200.1	*CsCKX1*	CsCKX1	547	61.96	9.10	37.48	93.73	−0.090	Vacuole
CsaV3_4G036030.1	*CsCKX2*	CsCKX2	518	57.37	5.27	33.75	96.12	−0.077	Extracellular
CsaV3_4G027750.1	*CsCKX3*	CsCKX3	699	79.32	8.52	34.02	96.38	0.027	Vacuole
CsaV3_3G040790.1	*CsCKX4*	CsCKX4	547	62.00	7.05	36.88	91.26	−0.214	Vacuole
CsaV3_2G029070.1	*CsCKX5*	CsCKX5	545	60.77	5.87	33.29	89.41	−0.241	Extracellular
CsaV3_2G001450.1	*CsCKX6*	CsCKX6	526	59.92	8.10	42.00	84.24	−0.312	Extracellular
CsaV3_1G040510.1	*CsCKX7*	CsCKX7	518	58.06	6.24	35.17	91.04	−0.036	Extracellular
CsaV3_1G040500.1	*CsCKX8*	CsCKX8	516	58.15	6.21	35.84	88.20	−0.231	Extracellular
CsaV3_6G003310.1	*CsIPT1*	CsIPT1	320	35.80	9.00	49.55	92.62	−0.163	Chloroplast
CsaV3_5G034390.1	*CsIPT2*	CsIPT2	350	39.38	8.74	47.48	86.37	−0.278	Mitochondrion
CsaV3_7G028650.1	*CsIPT3*	CsIPT3	330	37.05	8.91	36.40	90.73	−0.204	Chloroplast
CsaV3_7G023250.1	*CsIPT4*	CsIPT4	323	36.30	5.71	33.53	89.04	−0.352	Mitochondrion
CsaV3_6G018570.1	*CsIPT5*	CsIPT5	326	36.85	8.60	37.94	92.36	−0.222	Mitochondrion
CsaV3_1G014570.1	*CsIPT6*	CsIPT6	474	53.14	6.44	37.58	85.93	−0.380	Chloroplast
CsaV3_3G013770.1	*CsIPT7*	CsIPT7	324	36.51	6.56	39.09	95.96	−0.219	Chloroplast, mitochondrion, peroxisome
CsaV3_4G008350.1	*CsIPT8*	CsIPT8	390	44.66	6.05	53.17	71.08	−0.564	Chloroplast, cytoplasm, mitochondrion, peroxisome

**Table 3:** Secondary structure of CsCKX and CsIPT proteins.

Protein Name	Sequence Length	*α*-Helix/%	*β*-Turn/%	Random Coil/%	Extended Strand/%
CsCKX1	547	34.55	7.13	40.77	17.55
CsCKX2	518	33.78	5.41	41.89	18.92
CsCKX3	699	34.33	6.29	40.92	18.45
CsCKX4	547	31.44	4.94	45.52	18.10
CsCKX5	545	33.39	6.06	42.02	18.53
CsCKX6	526	34.60	5.70	41.06	18.63
CsCKX7	518	31.66	6.18	41.51	20.66
CsCKX8	516	35.85	6.01	41.09	17.05
CsIPT1	320	50.94	7.19	29.69	12.19
CsIPT2	350	46.29	6.00	35.14	12.57
CsIPT3	330	43.33	6.36	36.97	13.33
CsIPT4	323	56.35	7.74	25.39	10.53
CsIPT5	326	48.47	8.59	30.06	12.88
CsIPT6	474	52.11	4.43	33.1	10.34
CsIPT7	324	50.62	7.41	29.94	12.04
CsIPT8	390	50.51	4.87	34.10	10.51

## Data Availability

Data are contained within the article.
